# 经支气管超声引导针吸活检（EBUS-TBNA）在肺癌诊断及分期中的应用

**DOI:** 10.3779/j.issn.1009-3419.2010.05.08

**Published:** 2010-05-20

**Authors:** Devanand ANANTHAM, Mariko Siyue KOH, 燕 丁, 娟 南

**Affiliations:** 1 Department of Respiratory and Critical Care Medicine, Singapore General Hospital, Singapore; 2 天津医科大学总医院，天津市肺癌研究所，天津市肺癌转移与肿瘤微环境重点实验室

**Keywords:** 支气管超声, 肺癌, 经支气管针吸活检

## Abstract

经支气管超声引导针吸活检（endobronchial ultrasound guided tranbronchial needle aspiration, EBUS-TBNA）支气管镜具有一置入的微型超声探头，以便实现实时的TBNA活检。在肺癌中，EBUS-TBNA的敏感性为88%-90%，特异性为100%。受试者工作特征曲线下面积低于0.99时，测试效果良好。其诊断率显著优于传统盲法TBNA。然而，其假阴性率仍然很高，大约为20%。因此，阴性针吸结果需经纵隔镜检查、外科取样或临床随访证实。新辅助化疗后纵隔的再分期并不十分乐观，据报道，EBUS-TBNA的敏感性仅为76%，阴性预测值仅为20%。该方法亦已被成功用于获取气管旁及支气管周围区域原发肿瘤的活检标本，敏感性为82%-94%。EBUS-TBNA的优势在于其可在门诊中实施，患者仅需中度镇静，且无需电离辐射消毒。尽管在多数研究机构中EBUS-TBNA目前仍仅用于肿大淋巴结的靶向取样，但也有可能实现影像学检查正常的纵隔的完全分期。因此，如果设备及专家条件具备，EBUS-TBNA可作为肺癌诊断及有创分期的一线方案之一。

## 前言

之前，可曲支气管镜的使用受到内镜视野范围的限制。支气管内超声（endobronchial ultrasound, EBUS）是创新性的，它将内镜专家的视野延伸至气道壁外，并使气道旁及支气管周围结构的活检成为可能。纵隔和肺门淋巴结以及中央型胸部肿瘤为该项技术的理想靶标。经支气管超声引导针吸活检（endobronchial ultrasound guided tranbronchial needle aspiration, EBUS-TBNA）支气管镜是在专用可曲支气管镜上安置有一微型线性超声探头，以便实现实时的TBNA活检。这一设计进一步提高了诊断率。

该综述旨在描述EBUS-TBNA的技术现状，并评价其在肺癌中作为诊断及分期方法的作用。纵隔淋巴结的取样可进行组织学诊断并同时对疾病进行分期，而无需对原发肿瘤进行活检。因此，很难将诊断从分期中分离出来。现已发表的大多数资料聚焦非小细胞肺癌（nonsmall cell lung cancer, NSCLC），而小细胞肺癌的数据相对较少。

## EBUS-TBNA的技术现状

EBUS支气管镜的插入端外径为6.9 mm，顶端外径为6.7 mm，明显大于标准的可曲支气管镜。因此，需使用经口腔插管法，而非经鼻插管法。内镜的观察视角为35度（斜角），移动支气管镜可使操作者的观察视角得到补偿。插管时应特别注意，当探头到达声门下区域时通常仅可看到声带前端。由于内镜视野有限且倾斜，通常支气管检查会受限。因此，为了完成对整个气道的检查，亦需要进行标准的支气管镜检查。7.5 MHz超声传感器为凸型，具有与支气管镜长轴平行的50度的扇形视野，可探查深度为20 mm-50 mm。支气管镜具有一个可容纳专用活检针的内径为2.0 mm的操作通道。穿刺针大小为21 G或22 G。穿刺针的顶端具有多个小凹痕，以增强超声回音。

支气管插管后，EBUS-TBNA探头被置于目标淋巴结或气管旁肿瘤的适宜位置。探头可弯曲以与气道壁接触，然后在旋转过程中轻柔后退。通常，使用注入生理盐水的气囊以利于接触（图 1）。然而，若气道粘膜表面与探头接触良好，并非必须使用气囊。淋巴结与肿瘤为等回声（灰色），而血管无回声（黑色；图 2）。亦可采用彩色多普勒进行区分。

**1:**
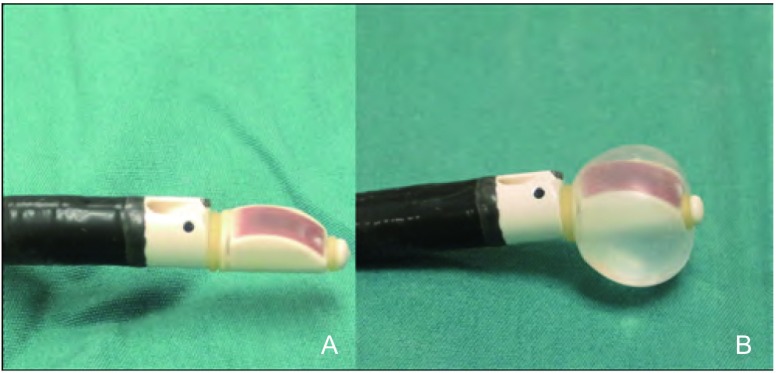
A:气囊内未注入生理盐水时的EBUS-TBNA支气管镜；B:气囊内注入1 mL-2 mL生理盐水时的EBUS-TBNA支气管镜。 A: EBUS-TBNA scope with balloon deflated. B: EBUS-TBNA scope with balloon inflated with 1-2 mL of saline.

**2:**
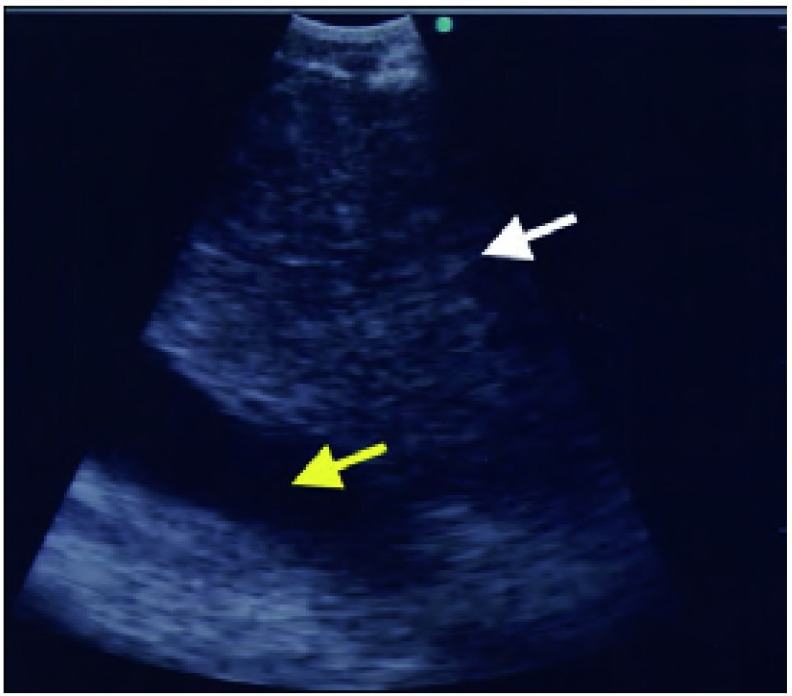
右气道旁淋巴结呈现圆形的等回声，如，灰色结构（白色箭头），上腔静脉呈现线性无回声，如，灰色结构（黄色箭头）。 Right paratracheal lymph node seen as a rounded isoechoic *i.e*. grey structure (white arrow) with the superior vena cava seen as a linear anechoic *i.e*. black structure deep to it (yellow arrow).

应用多普勒测定淋巴结的血管抵抗性指标的初步资料亦已出现^[1]^。由于新生血管较多，恶性淋巴结的抵抗性指标似乎更高。提示淋巴结恶性另一些特征包括：体积增大、圆形、内部的不均一性以及淋巴结结构的改变。当淋巴结结构改变时，正常情况下呈现高回声中心阴影的肺门淋巴结消失。

目标淋巴结或肿瘤一经确定，即可实施实时TBNA。实时指导下“猛刺术”（jabbing技术）用于TBNA之前，将针鞘向前推进以使其在内镜图像中可被观察到（图 3）。“猛刺术”为当操作者将穿刺针推进组织中时，助手应将支气管镜固定于患者口部。一旦TBNA穿刺针进入目标区内，在移动穿刺针以针吸活检之前，摇动穿刺针的口针以去除气道内的异物。在移动穿刺针约30 s后通过注射器进行抽吸。穿刺针的深度受位于一侧的钩扣的限制（图 4）。若不打开钩扣，穿刺的深度为17 mm。然而，去除这一限制后，穿刺可达粘膜下36 mm，达到更深的病灶。

**3:**
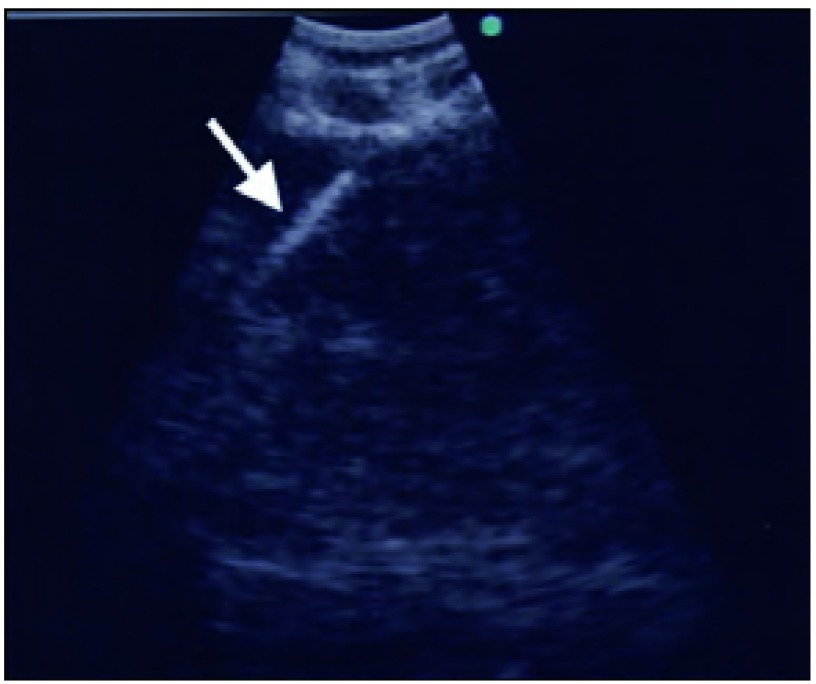
淋巴结内的实时EBUS-TBNA呈现高回声，如，白色穿刺针（白色箭头） Real-time EBUS-TBNA with hyperechoic *i.e*. white needle (white arrow) within a lymph node

**4:**
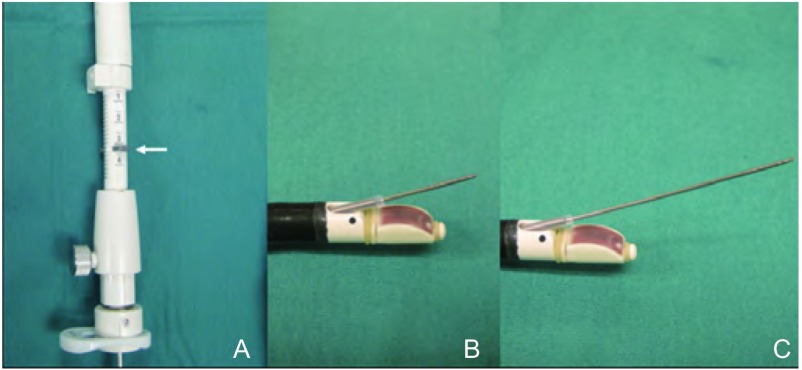
A:专用TBNA穿刺针（白色箭头）的安全钩扣；B：钩扣在原位时完全展开的穿刺针；C：钩扣释放时完全展开的穿刺针。 A: Safety catch on dedicated TBNA needle (white arrow). B: Needle in full extension with catch in place. C: Needle in full extension with catch released.

除主动脉-肺动脉（站5）、主动脉下（站6）、食管周围（站8）以及肺韧带（站9）淋巴结外，EBUSTBNA支气管镜可到达大多数的纵隔及肺门淋巴结。对于NSCLC的纵隔取样，推荐每一站淋巴结做3次细胞学穿刺。若获得了足够的芯样，2次取样即可^[2]^。首先从最高站的对侧淋巴结取样，取样顺序为N3、N2，最后为N1淋巴结。这可防止由于先前取样造成的穿刺针污染而使疾病分期增高。另外，在每一站淋巴结可使用新的针头，但这会使成本增加。

## NSCLC纵隔分期的现行指南

NSCLC的治疗选择由疾病的分期决定。转移性N2或N3纵隔淋巴结肿大不宜选择手术切除，而应选择化放疗^[3]^。无创分期通常是对这些患者进行评估的开始。

计算机断层（computed tomography, CT）扫描可提供肺癌的解剖学情况。然而，其识别纵隔淋巴结转移的总敏感性和特异性分别为51%和85%^[4]^。正电子发射计算机断层显像（positron emission tomography, PET）更为精确，其敏感性和特异性分别为74%和85%^[4]^。这些无创检查的阳性预测值得不确定意味着明确治疗前对纵隔异常的组织学检查必须持谨慎态度。唯一的例外可能是X线检查为广泛性纵隔浸润的患者，提示不可切除的疾病^[4]^。因此，美国胸科医师协会循证临床实践指南推荐，不论PET扫描结果为阳性或阴性，伴有弥散性纵隔淋巴结的患者均应进行淋巴结转移的组织学证实^[5]^。

纵隔镜已被认为是组织学分期的参考标准。该方法的总的诊断敏感性为80%，假阴性率为10%^[5]^。尽管纵隔镜易于对隆突前及气管旁病灶进行取样，但在对气管后及纵隔下淋巴结站进行取样时存在很多限制。其亦为一种需全身麻醉的有创方法，据报道其并发症的发生率为2%-3%^[6]^。而且，2001年临床实践调查显示仅27%的肺癌患者采用纵隔镜检查进行术前分期^[7]^。在采用纵隔镜检查的患者中，仅47%患者的淋巴组织为病理学诊断提供了证据，提示活检标本并非淋巴结站的标本^[7]^。这些警示信息提示需要一种引导的且简单易行的纵隔分期方法。因此，现行指南推荐，根据现有的设备和专家，纵隔镜检查或穿刺技术均可作为纵隔有创分期的合理方法^[4]^。穿刺技术包括传统的TBNA、EBUS-TBNA、内镜超声（endoscopic ultrasound, EUS）以及经胸壁针吸活检。

## EBUS-TBNA在纵隔分期中的应用

EBUS-TBNA在肺癌中的总的诊断敏感性为88%-90%，特异性为100%。然而，其假阴性率较高，约为20%^[5, 8]^。假阴性率由纳入汇总分析的各项研究所涉及的恶性肿瘤的高发病率引起。受试者工作特征曲线面积在0.99以下时，EBUS-TBNA测试效果较好^[8]^。其诊断率明显优于传统盲法TBNA^[9]^。研究表明，与无创方法如CT、PET或CT/PET相比，EBUS-TBNA可导致超过25%的患者的淋巴结分期升高或降低^[10]^。现有文献共报道了2 500余例病例，其中多数文献的目的是对肿大淋巴结进行病理分期（表 1）。已有数据的联合分析受恶性肿瘤不同的发病率、目标淋巴结的大小、术前显像如PET的使用、穿刺的次数、内镜专家的水平以及现场细胞学检查获得情况的影响。而且，所列出的20项研究中的9项研究共涉及1 400余例患者，该9项研究由两个专家合作组实施：来自海德堡/波士顿的Herth/Ernst等^[11-15]^以及来自日本的Yasafuku/Nakajima等^[16-19]^。

**1:** EBUS-TBNA对疑似肺癌患者纵隔及肺门淋巴结取样的诊断敏感性及明确的组织学诊断率 Diagnostic sensitivity and definitive histological yield of EBUS-TBNA sampling of mediastinal and hilar lymph nodes in patients with suspected lung cancer

研究	病例数（*n*）	恶性肿瘤的发病率（%）	淋巴结大小（mm）	诊断敏感性（%）	明确的组织学诊断率（%）
Lee HS等^[2]^	102	31	5-20	94	29
Wallace MB等^[8]^	138	30	-	69	21
Vincent BD等^[9]^	152	74	-	99	40
Herth F等^[10]^	502	98	8–32	94	94
Herth F等^[11]^	100	21	4–10	92	19
Herth F等^[12]^	100	9	5-10 PET阴性	89	8
Ernst A等^[13]^	66	89	10-21	87	59
Ernst A等^[14]^	213	88	8-20	91	-
Yasufuku K等^[15]^	70	67	≤30	96	71
Yasufuku K等^[16]^	108	63	8–30	95	59
Yasufuku K等^[17]^	102	25	5–22	92	24
Nakajima T等^[19]^	43	58	3-35	92	54
Feller-Kopman等^[22]^	135	35	-	85	44
Bauwens O等^[38]^	106	58	5-40 PET阳性	95	56
Rintoul RC等^[44]^	18	58	6-20	85	65
Szlubowski A等^[45]^	226	64	13.8±9	89	57
Hwangbo B等^[46]^	117	26	5-20	90	23
Rintoul RC等^[47]^	109	77	PET阳性	91	71
Vilmann P等^[48]^	33	65	-	85	47
Ømark Petersen H等^[49]^	157	43	> 10或PET阳性	85	36
Huangbo B等^[50]^	117	100	5-20	90	23
Reprinted with permission from the copyright holder ©Tianjin Lung Cancer Institute and Blackwell Publishing Asia Pty. Ltd 本表得到版权所有者©天津市肺癌研究所和Wiley-Blackwell复制许可					

EBUS-TBNA得出确切诊断的能力与恶性肿瘤的发病率有关（表 1）。阴性活检须经纵隔镜、外科淋巴结取样或临床随访证实，以弥补该技术阴性预测值的局限。因此，NSCLC的有创分期指南推荐源自穿刺技术（包括EBUS）的阴性活检结果须经纵隔镜进一步证实^[5]^。

一项以外科切除作为金标准的比较研究发现，实时EBUS-TBNA与经颈纵隔镜检查在敏感性方面具有相同的诊断率^[13]^。这项“头对头”（head-to-head）研究受一事实的限制：所有活检的淋巴结均肿大（10 mm-20 mm）。非肿大淋巴结是否可得到相似的数据仍需证实。对淋巴结肿大≤10 mm或PET检查为阴性的影像学检查正常的纵隔进行组织学分期是可行的，其敏感性接近90%^[12, 13]^。这些数据可能提示，与目前广泛开展的靶向取样相比，系统分期是可行的。然而，鉴于EBUS-TBNA通常在中度镇静的情况下实施，而这两项研究为专家在全身麻醉状态下实施，故这些数据的广泛适用性尚不明确。但EBUS可能会识别出CT未能显像的肿大淋巴结^[20]^。

据报道，即使未进行快速的现场细胞学检查，实时EBUS-TBNA的诊断率仍>90%^[11]^。如可进行现场细胞学检查，适量的淋巴细胞（>40/每功率场）或黑色素巨噬细胞为淋巴结取样充足的标志^[21]^。如疑似为淋巴瘤或肉芽肿，现场细胞学检查也可指导TBNA针吸活检后的进一步检查，如流式细胞术或微生物培养^[22]^。

EBUS-TBNA在诊断鳞癌（敏感性为85.7%）与腺癌（敏感性为90%）方面无明显差别^[23]^。专用22 G EBUSTBNA穿刺针获取的样本足以进行遗传学及分子分析（如，表皮生长因子受体突变）^[24, 25]^。该信息可指导制定生物制剂如吉非替尼的治疗计划。据报道，EBUSTBNA用于联合化疗后纵隔再分期的效果并不理想，诊断敏感性仅为76%，阴性预测值为20%^[26]^。因此，分期方案通常涉及用于初始分期的针吸活检以及再分期的纵隔镜检查。

EBUS-TBNA与经食管EUS引导的针吸活检联合可实现纵隔的完全分期，并可到达任一技术本身均不可到达的淋巴结站。EUS可到达主动脉肺动脉（站5）、食管周围（站8）以及肺韧带（站9）淋巴结站，而EBUS-TBNA均不可到达。EBUS更易于到达气道旁和肺门淋巴结站，而EUS可靶向作用于下纵隔淋巴结以及肾上腺转移灶。EUS在纵隔分期中的总诊断敏感性为84%^[5]^，而据报道，EBUS与EUS联合的诊断敏感性为93%-94%^[9, 27]^。然而，该联合分期的主要缺点为胃肠病专家和肺脏专家需协同操作。熟练掌握这两项技术的内镜专家极少。为了克服这一难题，目前通过使用EBUS支气管镜而开展经食管针吸活检实属创举^[28]^。通过单个探头，先实施EBUS-TBNA而后实施经食管EBUS-TBNA可完成分期。

EBUS-TBNA可成功获取位于气道旁及支气管周围区域的原发肿瘤的活检标本，诊断敏感性为82%-94%^[29, 30]^。若肺组织无充气，超声很容易将肿瘤识别为软组织结构。该技术与淋巴结取样相似，尤其有助于诊断未累及气道的中央型肿瘤。

## EBUS-TBNA在其它疾病诊断中的应用

一项单一回顾性研究显示EBUS-TBNA对临床疑似的淋巴瘤患者的诊断敏感性为91% ^[31]^。在诊断非干酪性肉芽肿性炎症时，EBUS-TBNA对结节病的诊断率为83%-94%^[32-36]^。一项随机对照试验显示EBUS-TBNA较标准TBNA更为有效^[35]^。除肺癌外，如组织学分析需要更大的组织标本，可通过EBUS探头插入一个1.5 mm的活检钳，经由穿刺针穿刺使其穿过气道壁。这样可获得纵隔病灶的实时活检^[35]^。

## EBUS-TBNA服务的建立

EBUS相关的培训包括图像判读、超声处理控制按钮的使用、内镜及超声视野的定位以及TBNA技术。胸外科解剖学知识及基本可曲支气管镜的熟练应用为先决条件。目前尚无有关EBUS-TBNA练习的监督次数的指南。然而，数据表明仅10次操作后，诊断的正确率可由50%提升至96%^[38]^。对于肿大纵隔淋巴结的靶向取样来说，10次训练即可，然而掌握非肿大淋巴结的完全分期和系统取样可能需要更长时间。另一项研究显示经过仅50次操作后诊断正确率即达高峰^[39]^。

EBUS-TBNA直接相关的并发症较为罕见。据报道，伴有潜在慢性阻塞性肺疾病的患者可发生气胸，需植入胸管^[40]^。其它情况下仅会出现一些轻微的并发症：焦虑、咳嗽以及穿刺部位的短暂出血^[16, 41]^。由于可通过小心地穿过肺动脉进行活检，所以不慎穿刺血管而导致出血的风险很小^[42]^。

当支气管镜经过口咽部时，TBNA穿刺针可能会被共生物污染。这可能会引起肺、纵隔或心包的感染^[43]^。研究认为，当TBNA穿刺针被完全展开（36 mm）时，风险可能增加^[43]^。在这一深度，无法通过超声清晰地看到穿刺针的顶端。然而，菌血症的发生风险与常规支气管镜相似，且并无证据支持常规使用预防性抗生素^[44]^。

EBUS可为内镜检查提供充分的时间，且可在中度镇静情况下进行操作。据报道，在熟练操作的情况下，利用EBUS-TBNA对靶向淋巴结的取样平均需12.5 min（8 min-21 min）^[11]^。然而，进行完全纵隔分期可能会需要更长时间。

使用活检穿刺针对经导管进行穿刺时可能会损伤支气管镜。实施TBNA前通过内镜视野观察针鞘可预防该损伤。需要强调的另外一些问题为，培训内镜专业人员重新学习EBUS支气管镜并在操作过程中提供帮助。在移去气囊时，尖锐的指甲很容易划伤敏感的超声传感器。在服务建立之前，需权衡设备及一次性物品的补偿与消耗。

## 结论

研究已证实，EBUS-TBNA在获取肺癌患者的组织学标本方面具有较高的诊断率。作为诊断方法，其不失为中央型气管周围肿瘤及纵隔淋巴结肿大患者的理想选择。无需电离辐射消毒即可“透过气管壁探查病灶”的能力是EBUS发展的前提。现有数据表明，在设备及专家条件具备的情况下，EBUS-TBNA为NSCLC有创分期诊断的一线手段之一。尽管在多数研究机构中EBUS-TBNA目前仍仅用于肿大淋巴结的靶向取样，但也有可能实现影像学检查正常的纵隔的完全分期。

EBUS的并发症较少、诊断率的较高以及学习曲线较短使其有可能成为标准支气管镜检查的一部分。EBUS-TBNA亦可通过几种途径节省费用^[43]^。较高的诊断率意味着很少需要重复检查或采用另一种检测方法。其可在门诊中实施，患者仅需中度镇静，因此避免了住院花费。通过准确的纵隔分期，“不必要的”纵隔镜检查或外科切除亦可避免^[9, 40, 41]^。今后的研究应着重基于具有声像学特点的特征化的淋巴结，以更好地靶向作用于病变淋巴结。在不久的将来，设备的改进及训练机会的增加将会使该技术更为广泛应用和接受。
